# PERCEIVED AND PHYSIOLOGICAL STRAINS OF SOCIETAL PARTICIPATION IN PEOPLE WITH MULTIPLE SCLEROSIS: A REAL-TIME ASSESSMENT STUDY

**DOI:** 10.2340/jrm.v56.40838

**Published:** 2024-06-24

**Authors:** Arianne S. GRAVESTEIJN, Maaike OUWERKERK, Isaline C.J.M. EIJSSEN, Heleen BECKERMAN, Vincent DE GROOT

**Affiliations:** 1 MS Center Amsterdam, Rehabilitation Medicine, Vrije Universiteit Amsterdam, Amsterdam UMC location VUmc, Amsterdam; 2 Amsterdam Neuroscience Research Institute, Neuroinfection & Neuroinflammation, Amsterdam; 3 Amsterdam Public Health Research Institute, Social Participation and Health, Amsterdam, The Netherlands

**Keywords:** activities of daily living, digital health, heart rate, multiple sclerosis, patient-reported outcome measure, societal participation

## Abstract

**Objective:**

To examine the relationship between perceived and physiological strains of real-time societal participation in people with multiple sclerosis.

**Design:**

Observational study.

**Subjects/Patients:**

70 people with multiple sclerosis.

**Methods:**

Perceived and physiological strain of societal participation (10 participation-at-location and 9 transport domains) were measured in real time using the Whereabouts smartphone app and Fitbit over 7 consecutive days. Longitudinal relationships between perceived (1 not strenuous to 10 most strenuous) and physiological strains (heart rate reserve) were examined using mixed-model analyses. Type of event (participation-at-location or transport) was added as covariate, with further adjustments for fatigue and walking ability.

**Results:**

Median perceived strain, summarized for all societal participation domains, varied between 3 and 6 (range: 1–10), whereas physiological strain varied between 18.5% and 33.2% heart rate reserve. Perceived strain (outcome) and physiological strain were not associated (β -0.001, 95%CI -0.008; 0.005, with a 7-day longitudinal correlation coefficient of -0.001). Transport domains were perceived as less strenuous (β -0.80, 95%CI -0.92; -0.68). Higher fatigue levels resulted in higher perceived strain (all societal participation domains) (β 0.05, 95%CI 0.02; 0.08).

**Conclusion:**

Societal participation resulted in low-to-moderate perceived and physiological strain. Perceived and physiological strain of societal participation were unrelated and should be considered different constructs in multiple sclerosis.

LAY ABSTRACT

People living with multiple sclerosis often experience a wide variety of symptoms, such as muscle weakness and fatigue, which can result in high patient-perceived strain during daily living. Moreover, in research settings activities of daily living require more energy in people with multiple sclerosis when compared with healthy peers. During physical exercise the energy requirements are closely related to patient-perceived strain, but whether this is also the case in real-time daily living is unknown. Therefore, we examined the relationship between patient-perceived strain and energy requirements during real-time societal participation. With the Whereabouts smartphone application and Fitbit, we were able to measure both patient-perceived strain and energy demands. In 70 people living with multiple sclerosis, we found that these measures were unrelated. The level of fatigue played an important role in this relationship; a higher level of fatigue resulted in higher patient-perceived strain.

Multiple sclerosis (MS) is characterized by time-variable motor, sensory, visual, emotional, and cognitive impairments, resulting in an unpredictable disease course for most people living with MS (pwMS) (1). These impairments restrict pwMS in social functioning and societal participation (2).

The International Classification of Functioning, Disability and Health of the World Health Organization defines participation as “involvement in life situations” (3). Participation not only depends on the ability to perform activities, but also entails well-being, meaningfulness, satisfaction, and the ability to perform social roles in a social environment (4–9). Active societal participation is essential for personal well-being and closely linked to quality of life (4, 10, 11). These concepts are linked to the definition of health introduced in 2011: “The ability to adapt and to self-manage in the face of social, physical, and emotional challenges” (12). Improving societal participation is therefore an important treatment goal in rehabilitation medicine (2, 4).

While it is essential to measure societal participation, selecting and carrying out appropriate measurements presents a complex challenge (6, 13). Current measures of societal participation fail to take into account real-time assessment, perceived experiences, and meaningfulness (2). Each person has a unique experience of societal participation, determined by individual social roles and cultural and political environments (2). The need for an assessment tool that takes into account a person’s perceived, unique, real-time assessed societal participation has led to the development of the “Whereabouts” smartphone application (14).

The strain of societal participation can be expressed as perceived strain (a person’s self-perceived physical and/or psychological effort (15)). Strain can also be expressed quantitatively as physiological strain; for example, as percentage heart rate reserve (%HRR) (16). Since pwMS often experience motor problems and fatigue (1, 17), perceived strain might be high and become a limiting factor for societal participation. Ainsworth and colleagues described the physiological strain for a variety of activities of daily living in healthy individuals (18). However, studies indicate that people with disabilities may consume more energy than healthy people when engaged in societal participation (19, 20), which suggests that monitoring both perceived and physiological strain in pwMS is relevant.

Previous literature demonstrated a strong positive relationship between perceived and physiological strain during exercise in healthy individuals and in pwMS (21, 22). However, to our knowledge the relationship between perceived and physiological strain of societal participation in pwMS has not yet been examined. Better insight into this relationship during societal participation can help elucidate the high perceived strains during daily living in pwMS.

The aim of this study was to determine the relationship between perceived strain and physiological strain during societal participation in pwMS. We hypothesized that perceived and physiological strain are positively associated. Due to mobility problems and MS-related fatigue, physical strain values are expected to be higher compared with the general population (19, 20, 23). If such a positive relationship exists, the Whereabouts app can be helpful in correctly assessing and interpreting strains, as well as in the efficient tailoring of self-management programs and other rehabilitation treatment programs for pwMS.

## METHODS

### Study design and participants

Participants were recruited at the department of Rehabilitation Medicine, Amsterdam UMC, location VUmc, the MS Center Amsterdam, and through local advertisement (i.e., website local MS organizations and information folder). PwMS that showed interest received a study information letter containing information on the rationale, purpose, and duration of the study. Participants were eligible if they had a definite diagnosis of MS confirmed by a neurologist, were at least 18 years old, and possessed a smartphone (Android [5+] or IOS [9+]). People were excluded if they lived in a nursing home or received inpatient care, were incompetent to give informed consent, or if they reported that they cannot use a smartphone very well to answer app-related questions. After providing written informed consent, pwMS were screened by telephone for inclusion and exclusion criteria. Baseline home visits were scheduled for the installation of the Whereabouts smartphone app (see below) and participants were instructed on how to use both the app and Fitbit. The Whereabouts smartphone app and Fitbit were used for 7 consecutive days. Prior to baseline visits at home, participants filled in questionnaires to determine age, gender, disease duration, type of MS, fatigue with the Checklist Individual Strength subscale fatigue (CIS20r fatigue), walking ability assessed with the Multiple Sclerosis Walking Scale (MSWS) (24, 25), and anxiety and depression measured with the Hospital Anxiety and Depression Scale (HADS).

Ethical approval was waived by the Medial Ethics committee of Amsterdam UMC, location VUmc (reference number: 2018.677, date: 10 January 2019). The study was conducted in accordance with the Declaration of Helsinki.

### Sample size

Sample size was set at 80 pwMS, taking into account an eventual dropout of 15%, to investigate the validity of the Whereabouts app with adequate statistical power. A sample size of 70 participants is well above the recommended minimum of *n* = 50 to achieve adequate statistical power in clinimetric studies (14, 26, 27).

### Whereabouts smartphone app

The Whereabouts smartphone app was developed to determine meaningful societal participation. The application automatically and accurately measures real-time location and transport using GPS (14). Participants were asked to define the type of participation performed at a location as well as the type of transport used between locations from a pre-specified list (Table I). Real-time segments are created at a location or during transport. After each time segment, the Whereabouts smartphone app automatically asks users for information about the type of participation or transport, meaningfulness, and strain. Meaningfulness and strain were measured using 1- to 10-point scales, 1 indicating not meaningful to me at all and 10 very meaningful to me, and 1 not strenuous at all and 10 most strenuous, respectively. It was explained to participants that strain was considered both mental and physical strain.

**Table I:** Societal participation domains

Types of societal participation	Transport means
1. Personal care (washing, dressing, eating, drinking, sleeping)	1. Walking
2. Household tasks, groceries, gardening	2. Bicycle, hand-bike
3. Work, education	3. Mobility scooter
4. Providing care for others	4. Wheelchair (manual or electronic)
5. Carrying out daily routine (planning and organizing of daily living and managing expenses)	5. Car as driver
6. Healthcare utilization	6. Car as passenger (incl. taxi, supplementary public transport)
7. Recreation, leisure, sport	7. Public transport (train, bus, tram)
8. Social interaction and relationships	8. Electric bike
9. In transit	9. Other
10. Other	

### Fitbit

Heart rate as a measure of physiological strain was assessed continuously (i.e., day and night) by a Fitbit Charge 2 or 3 during use of the Whereabouts smartphone app (i.e., 7 consecutive days), except for a 1- to 2-hour period after day 4 when the Fitbit needed to be recharged and was not worn. The Fitbit measures heart rate continuously using PurePulse, a photoplethysmography technique (28). The Fitbit Charge is a valid measurement tool to determine heart rate during daily living conditions (29). However, recent findings do indicate a possible underestimation of heart rate determined with a Fitbit Charge (30).

### Data analysis

Perceived strain and start–stop times for the various segments of societal participation or transport were documented in the Whereabouts smartphone app (14). Fitbit data were downloaded from the Fitbit webpage. Per day a Fitbit .JSON-file for each participant was uploaded in Matlab (version R2016b Mathwork, Natick, MA, USA). Time points and heart rates for the 7 consecutive days were combined and visually assessed for continuous measurement. If participants did not have data covering the entire 7-day measurement period they were excluded from the analysis. To account for relative intensities of the different societal participation and transport segments, heart rate reserve (%HRR) (calculation I) was determined as a measure of physiological strain (16).

Calculation I: %HRR = (HRtask-HRrest)/(HRmax-HRrest)

HRtask is the mean heart rate during a societal participation segment at location or during transport, HRrest is the lowest mean heart rate over 1,000 consecutive heart rate measures during the 7 consecutive days, and HRmax is the predicted maximal heart rate based on the Karvonen formula: 220 – age (31).

For all societal participation at location and transport segments, start and stop dates and times were registered by the Whereabouts smartphone app as dd/mm/yyyy, hh:mm:ss in central European summer time. In Matlab these time points were synchronized to the same time points in the Fitbit data (i.e., dd/mm/yyyy, hh:mm:ss in central European summer time). Mean heart rate and standard deviation of heart rate were established for each segment (i.e., from start to stop). Finally, %HRR was calculated for all societal participation segments.

### Statistical analysis

Statistical analysis was performed using STATA 14 statistical software (StataCorp LLC, College Station, TX, USA). Normality of distributions was checked by visual assessment of histograms.

The number of accurately measured perceived and physiological strain values was determined for all societal participation segments. Physiological strain (i.e., %HRR) is presented as mean (SD) and perceived strain is presented as median (range). The number of participants that contributed to the different domains is also reported.

The longitudinal relationship between participant-reported perceived strain in the Whereabouts smartphone app (i.e., dependent variable) and physiological strain of societal participation as measured by the Fitbit, operationalized by %HRR (i.e., independent variable), was assessed with linear mixed-model analysis. The linear mixed model was built from simple regression to a random intercept model, followed by a random intercept–random slope model (32). Finally, type of event (i.e., societal participation at location = 0 or transport = 1) was added to the best fitted model, first as a determinant and then with an interaction term with %HRR. To adjust for possible effects of fatigue, walking ability, anxiety, and depression, the scores on the CIS20r fatigue, MSWS, and HADS subscales were added to the model. Fitting of the models was compared using the likelihood-ratio test (33). To better understand the strength of the relationship, analyses were repeated for the domain “recreation, leisure and sport”. In this domain, which consists of intensive sports activities but also relaxation during leisure time, a wide variance between and within participants was expected. This could potentially lead to a stronger relationship between perceived and physiological strain.

A longitudinal correlation coefficient was calculated to determine the correlation between perceived and physiological strain. The longitudinal correlation coefficient was determined for the best fitted models by multiplying the regression coefficient of that model by the SD of the independent variable (%HRR) and dividing this by the SD of the dependent variable (perceived strain). The correlation was determined to be weak, moderate, or strong in case of a correlation < 0.3, 0.3–0.7, or > 0.7, respectively (34).

## RESULTS

### Participants

In total, 77 pwMS were included in the study. Seven individuals were subsequently excluded from analyses because data from the app (*n* = 4) or Fitbit (*n* = 2) were not available due to technical difficulties or the participant was lost to follow-up (*n* = 1). The remaining 70 participants had a mean age of 51 years (ranging from 25 to 72 years of age), a mean disease duration of 11 years, a median MSWS score of 40%, and the most common disease subtype was relapsing remitting MS (Table II).

**Table II:** Sociodemographic and disease-related characteristics of people living with MS (pwMS) included in the analysis

Characteristics	*n* = 70
Age in years, mean (SD)	50.7 (10.4)
Gender, *n* (%)	
Female	55 (78.6)
Male	14 (20.0)
Gender neutral	1 (1.4)
Disease duration in years, median (IQR)	11 (6–20)
Type of MS, *n* (%)	
Relapsing remitting MS	45 (64.3)
Secondary progressive MS	12 (17.1)
Primary progressive MS	8 (11.4)
Unknown	5 (7.1)
MSWS, median (IQR)	35.4 (14.6-66.7)
CIS20r fatigue, mean (SD)	35.2 (11.7)
HADS, median (IQR)	
Anxiety	5 (2–6)
Depression	3 (1–5)

SD: standard deviation; IQR: interquartile range; MS: multiple sclerosis; MSWS: Multiple Sclerosis Walking Scale; CIS20r: Checklist Individual Strength fatigue scores range between 8 (no fatigue) and 56 (very severe fatigue); HADS: Hospital Anxiety and Depression Scale.

### Perceived and physiological strain segments

**Fig. 1:**
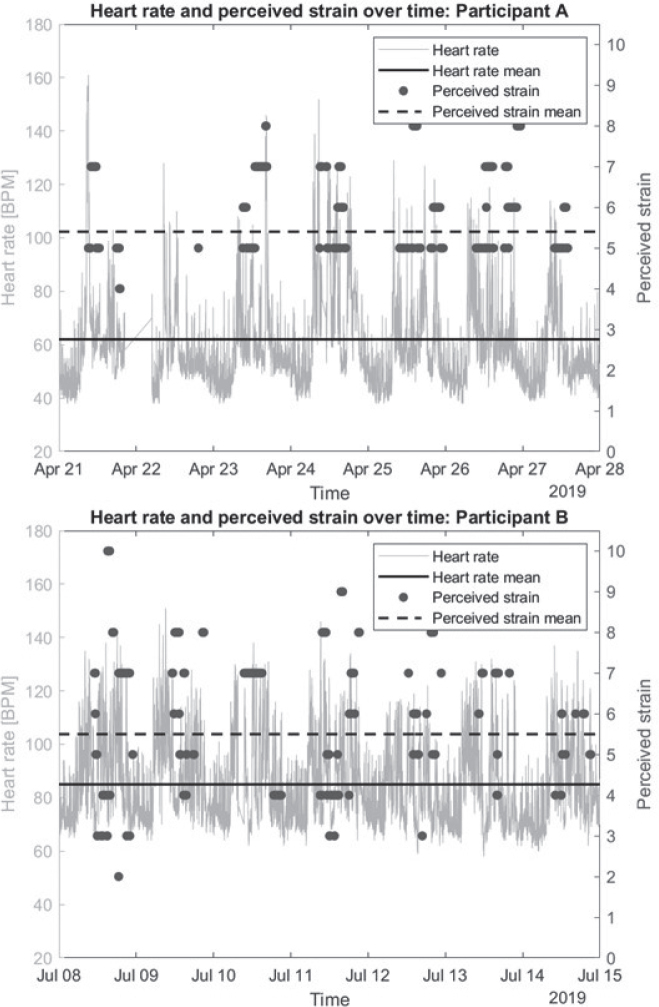
Perceived strain and heart rate coupling for 2 participants.

A total of 3,280 societal participation segments (at location or transport) were registered by the Whereabouts smartphone app for the 70 participants combined. Perceived strain values were missing on 208 occasions in 20 participants, and 142 heart rate measures in 34 participants were missing due to an interruption of the Fitbit, including 9 participants for whom perceived strain values and heart rate measures were both missing. A total of 2,939 outdoor participation (1,146 at location and 1,776 during transport, 17 undetermined event type) events were coupled and analysed. In Fig. 1, continuous heart rate measurement (grey line) and perceived strain during societal participation segments (dark grey dots) are plotted for 2 participants, demonstrating differences between individuals regarding perceived strains (in participant A varying from 4 to 9 and in participant B from 2 to 10) and heart rate (participant A shows greater variability in heart rate than participant B).

### Perceived and physiological strains of societal participation

Median perceived strain and mean physiological strain values for the participation at location and transport domains are presented in Table III. The number of registered segments per domain, as well as number of participants who contributed to these domains, are also presented in Table III.

**Table III:** Patient-perceived and physiological strain of participation at location and transport domains

Factor	Perceived strain			Physiological strain				Combined	
	Participants *n*	Perceived strain segments *n* (missing)	Perceived strain Median (range)	Participants *n*	Heart rate segments *n* (missing)	Heart rate reserve %, mean (SD)	Heart rate Mean (SD)	Participants *n*	Heart rate & perceived strain sets *n* (missing)
Participation at location									
Personal care	34	51 (7)	5 (1–9)	34	56 (2)	25.00 (10.35)	85.76 (12.22)	34	50 (8)
Household tasks, groceries, gardening	61	330 (1)	5 (1–10)	59	316 (15)	21.46 (9.73)	82.98 (13.11)	59	315 (16)
Work or Education	36	149 (22)	5 (1–10)	36	170 (1)	22.10 (8.51)	84.17 (10.08)	36	148 (23)
Providing care for others	36	84 (3)	5 (1–10)	34	83 (4)	22.39 (8.83)	82.93 (12.02)	34	80 (7)
Carrying out daily routine	27	57 (0)	5 (1–10)	26	53 (4)	21.84 (8.96)	80.53 (12.16)	26	53 (4)
Healthcare utilization	31	68 (6)	5.5 (1–10)	31	72 (2)	21.31 (8.05)	81.18 (10.08)	31	66 (8)
Recreation, leisure and sports	53	208 (6)	5 (1–10)	54	198 (16)	23.97 (10.86)	85.06 (14.19)	53	192 (22)
Social interactions and relationships	54	186 (5)	5 (1–9)	52	186 (5)	21.52 (8.49)	81.11 (12.02)	52	181 (10)
In transit	14	42 (0)	5 (1–10)	13	41 (1)	15.84 (7.10)	75.56 (9.56)	13	41 (1)
Other	11	20 (2)	5 (1–9)	11	22 (0)	18.50 (5.45)	76.52 (8.74)	11	20 (2)
Total		1,195 (52)			1,197 (50)				1,146 (101)
Transport domains									
Walking	52	359 (37)	5 (1–9)	52	376 (20)	21.98 (10.80)	83.19 (13.01)	52	341 (55)
Bicycle, hand-bike	39	353 (0)	5 (1–9)	38	338 (15)	22.08 (11.42)	80.45 (14.61)	38	338 (15)
Mobility scooter	10	166 (0)	3 (1–8)	10	164 (2)	19.44 (11.45)	79.04 (14.85)	10	164 (2)
Wheelchair	11	34 (0)	6 (1–9)	11	33 (1)	18.61 (13.06)	88.05 (12.01)	11	33 (1)
Car as driver	53	611 (11)	3 (1–8)	53	587 (35)	21.64 (9.84)	82.84 (12.50)	53	576 (46)
Car as passenger (incl. taxi and supplementary public transport)	42	243 (6)	4 (1–8)	42	239 (10)	20.52 (8.47)	84.53 (11.00)	42	233 (16)
Public transport (train, bus, tram)	13	74 (12)	5 (2–9)	13	85 (1)	19.16 (9.27)	79.89 (13.42)	13	73 (13)
Electric bike	1	10 (3)	2 (2–5)	3	12 (1)	23.06 (9.60)	90.53 (9.85)	1	10 (3)
Other	3	9 (0)	2 (1–7)	3	8 (1)	33.17 (12.52)	94.90 (11.25)	3	8 (1)
Total		1,859 (69)			1,842 (86)				1,776 (152)

SD: standard deviation.

Overall, the median perceived strain of societal participation domains varied between 3 and 6, with interpersonal ranges between 1 and 10. The mean physiological strain across the domains ranged from 18.6 to 33.2%HRR. The societal participation at location domain “other”, and transport domains “other” and “electric bike” were not taken into account, as these domains were not clearly described and the number of contributors was small (i.e., 3, 1, and 11, respectively). The domain “household tasks, groceries and gardening” was the most often reported participation at location, with a median perceived strain of 5, range 1–10 (330 segments in 61 participants), and mean physiological strain 21.5% HRR, SD 9.7 (316 segments in 59 participants). The highest reported median perceived strain was 5.5 (range: 1–10) for the domain “health care utilization”, whereas the median perceived strain for all other participation at location domains was 5. Conversely, the mean physiological strain for the domain “health care utilization” was 21.3% HRR, which was the third lowest physiological strain value. The highest reported physiological strain was measured for the domain “personal care” (mean 25.0%HRR, SD 10.35).

For transport, the domain “car as driver” was reported most frequently, with a median perceived strain of 3, range 1–8 (611 segments in 53 participants), and mean physiological strain of 21.6%HRR (587 segments in 53 participants). The highest perceived strain was registered in the domain “wheelchair” (median: 6, range: 1–9), while physiological strain was shown to be the lowest in this domain (mean 18.61%HRR, SD 13.06).

### Relationship between perceived and physiological strains of societal participation

The relationship between perceived and physiological strain (%HRR) during societal participation and transport events over the 7-day monitoring period, ignoring intra-person dependency in the data, was significant (β –0.01, 95% CI –0.02 to –0.002) (Table IV). By adding a random intercept per individual and adjusting for this dependency, the model improved significantly (χ^2^ (1) = 1,492.70, *p* < 0.01), but the relationship between perceived and physiological strain was no longer significant (β 0.0003, 95% CI –0.007 to 0.007). Adding random slopes did not lead to further improvement of the model (χ^2^ (2) = 1.32, *p* > 0.05).

**Table IV:** Linear mixed models with perceived strain (outcome) and physiological strain (determinant), (n = 2,939)

Factor	b	SE_b_	95% CI	
			Lower	Upper
1. Simple regression				
Intercept	4.82	0.10	4.63	5.01
% HRR	–0.01	0.004	–0.02	–0.002
2. Random intercept^a^				
Intercept	4.69	0.19	4.32	5.07
% HRR	0.0003	0.004	–0.007	0.007
3. Random intercept & random slope^b^				
Intercept	4.70	0.20	4.30	5.09
% HRR	0.00002	0.004	–0.007	0.008
4. Adding type of event as predictor to the random intercept model^c,d^				
Intercept	5.22	0.20	4.83	5.61
% HRR	–0.001	0.003	–0.008	0.005
Type of event (ref. societal participation at location = 0)	–0.80	0.06	–0.92	–0.68
5. Adding interaction term of type of event and %HRR to random intercept model^c^				
Intercept	5.14	0.22	4.71	5.56
% HRR	0.003	0.005	–0.008	0.01
Type of event (ref. societal participation activities = 0)	–0.68	0.15	–0.97	–0.38
%HRR *Type of event	–0.006	0.006	–0.02	0.007
6. Adjustment for fatigue (CIS20r) and walking ability (MSWS) added to random intercept model^c,e^				
Intercept	3.32	0.52	2.30	4.34
% HRR	–0.001	0.003	–0.008	0.005
Type of event (ref. societal participation activities = 0)	–0.80	0.06	–0.92	0.68
MSWS	0.003	0.006	–0.008	0.015
CIS20r	0.05	0.02	0.02	0.08

a Significant improvement of model; χ^2^ (1) = 1492.70, *p* < 0.01;

b no significant improvement of model; χ^2^ (2) = 1.32, *p* = 0.52;

c *n* = 2,922, 17 missing due to registration error in type of event;

d type of event is a significant predictor of perceived strain;

e significant improvement of model; χ^2^ (2) = 13.86, *p* < 0.01.

SE: standard error; HRR: heart rate reserve; ref: reference category; CIS20r: Checklist Individual Strength; MSWS: Multiple Sclerosis Walking Scale; CI: confidence interval.

Type of event (i.e., location = 0 [reference category] or transport = 1) was added to the random intercept model and found to be a significant covariate in the relationship between perceived and physiological strain (β –0.80, 95% CI –0.92 to –0.068). Type of event was not a significant effect modifier in the relationship between perceived and physiological strain (*p* = 0.37). Subsequently, the model was adjusted for CIS20r fatigue and walking ability measured by the MSWS, which led to a further improvement of the model (χ^2^ (2) = 13.86, *p* < 0.01). HADS scores did not improve the model, and were excluded. In this final model, the relationship between perceived strain and physiological strain was not significant (β –0.001, 95%CI –0.008 to 0.005). A higher fatigue level resulted in higher perceived strain (Table IV).

### Recreation, leisure, sports domain

The domain “recreation, leisure and sports” was one of the most popular domains, with 192 documented and coupled segments for 53 of the 70 participants. Separate analysis of this domain showed a non-significant relationship between perceived and physiological strain (β 0.009, 95% CI –0.02 to 0.04 and β 0.007, 95% CI –0.02 to 0.03 adjusted for fatigue and walking ability) (Table V).

**Table V:** Linear mixed models with perceived strain (outcome) and physiological strain (determinant) for the domain “recreation, sports and leisure” (*n* = 192)

Factor	b	SE_b_	95% CI	
			Lower	Upper
1. Simple regression				
Intercept	5.41	0.43	4.57	6.25
%HRR	-0.01	0.02	-0.04	0.02
2. Random intercept^a^				
Intercept	5.12	0.43	4.28	5.95
%HRR	0.009	0.01	-0.02	0.04
3. Adjustment for fatigue (CIS20r) and walking ability (MSWS) added to random intercept model^b^				
Intercept	2.86	0.87	1.15	4.57
% HRR	0.007	0.014	-0.02	0.04
MSWS	0.018	0.010	-0.001	0.036
CIS20r	0.04	0.02	-0.01	0.09

a Significant improvement in model; χ^2^ (1) = 53.64, *p* < 0.01;

b significant improvement in model; χ^2^ (2) = 10.58, *p* < 0.01.

SE: standard error; %HRR: % heart rate reserve; CIS20r: Checklist Individual Strength; MSWS: Multiple Sclerosis Walking Scale; CI: confidence interval.

### Longitudinal correlation between perceived and physiological strain during societal participation

The regression coefficient of the best fitting model (i.e., the random intercept model with β –0.001) was multiplied by the SD of physiological strain (SD = 9.969) and divided by the SD of perceived strain (SD = 2.220), resulting in a longitudinal correlation coefficient of –0.004 between perceived and physiological strains during societal participation in pwMS. Sub-analysis for the domain “recreation, leisure and sports” demonstrated a longitudinal correlation coefficient of 0.03. These results do not support a longitudinal correlation between perceived and physiological strain during societal participation or during recreation, leisure and sports.

## DISCUSSION

In this study the relationship between real-time assessed patient-perceived and physiological strain during societal participation was assessed in pwMS. In contrast to our expectations, we found no relationship between perceived and physiological strain during societal participation in pwMS. Moreover, the relationship in the more physically orientated domain “recreation, leisure and sports” was similar to the model including all societal participation domains. This suggests that perceived and physiological strain are unrelated during societal participation.

Perceived strain, effort as experienced by the patient, was measured on a 1- to 10-point scale, taking into account both the physical and mental effort required for societal participation (15). At each location and after each use of transport participants were asked to score their perceived strain, which gave a realistic societal participation timeline coupled to real-time assessment of perceived strain (14).

In this study, perceived strain for the various domains was rated low-to-moderate, with a median perceived strain of 5 in 12 out of 19 societal participation domains (35). Adjusting the model for fatigue and walking ability resulted in a better fit. In particular, fatigue significantly affected the relationship between perceived and physiological strain, as a 10-point increase in fatigue level resulted in a 0.5 increase in perceived strain. In pwMS, the perception of bodily sensations might be altered, which might have contributed to the higher perceived strain (36).

The light to very light (i.e., HRR ranging from 15.8% to 25.0%) physiological strains observed were unexpected (16), because people with disabilities such as MS usually consume more energy when engaging in activities (20, 37). However, the energy expenditure of daily activities is often measured in laboratory settings where participants are asked to perform standardized activities according to strict research protocols. In the current study, societal participation was measured in a community-based setting where pwMS likely regulate their energy requirements by performing activities at a low intensity or low pace to compensate for a lack of energy, especially in the case of those more severely affected by the disease (38, 39).

Inconsistent with our original hypothesis, the results indicate that perceived and physiological strain are two different constructs; for example, the reported perceived strain for the transport domain “car as driver” was 3, which was among the lowest reported values, while the calculated physiological strain in this domain was 22%HRR, one of the highest values for physiological strain. Such inconsistencies were also found for the participation at location domain “health care utilization”. Here the median perceived strain was 5.5, the highest rated participation at location domain, while the mean physiological strain was only 21.3% HRR, amongst the least strenuous of the societal participation domains.

In contrast to the lack of a significant relationship between perceived and physiological strains in pwMS in this study, healthy people show a strong correlation between perceived strain and heart rate during exercise (*r* = 0.74) (22). During exercise, pwMS reportedly show strong correlations between perceived strain and physiological strain as measured by oxygen uptake (*r* = 0.69) or workload (*r* = 0.70); however, the same study found only a weak negative correlation between perceived strain and heart rate (*r* = –0.28) (21). For comparison, we found a correlation coefficient of –0.004 in our complete sample and 0.03 in the domain “recreation, leisure and sports” in our study. Whereas the main focus during exercise is on physical strain, the Whereabouts smartphone app focuses on societal participation in which personal and environmental circumstances likely impact perceived strain. Therefore, in our study the perceived strain of societal participation in the community can be seen as a construct that measures a combination of perceived physical and mental strain.

Considering that physiological strain does not explain perceived strain, disentangling differences between the two during societal participation might have important diagnostic and treatment implications. Societal participation appears to be less physiologically demanding than expected, while it is still perceived as strenuous by patients living with MS. This might be explained by fatigue, walking ability, or other factors (i.e., cognitive demands, body mass index), but does suggest that the focus of treatment should be less concerned with physiologically related issues and more concerned with perceived strain and related factors.

The Whereabouts smartphone app is a unique smartphone application that allows real-time assessment of meaningfulness of societal participation as well as real-time assessment of perceived strains. Using GPS tracking, the smartphone app was able to clearly distinguish between societal participation domains and transport means (14). The Whereabouts app was valued as highly important by its users (14), and this app clearly represents a valuable new patient-reported outcome measure in the field of rehabilitation medicine.

Societal participation was subdivided into pre-specified domains. The domains “car as driver” and “car as passenger” are clearly defined, and the physiological strain associated with car driving was expected to be low. By contrast, domains such as “cycling” or “work & education” can encompass a broad range of different activities, and therefore physiological strains will vary greatly. Nonetheless, only minimal differences were noted for all societal participation domains combined and for the domain “recreation, leisure and sports” specifically.

### Study strengths and limitations

To our knowledge, this is the first study comparing patient-perceived and physiological strains of societal participation in a heterogeneous group of people living with MS in a community setting.

Some study limitations should be mentioned. First, although the Whereabouts smartphone app was able to make a clear distinction between the various domains of participation at location and during transport (14), it was difficult to determine specific strains due to the broad pre-specified domains (e.g., “recreation, leisure and sport”, “household tasks, groceries and gardening”, “wheelchair (manual or electronic)”). Second, heart rate might not be the most appropriate indicator of physiological strain, as it can also fluctuate due to factors such as stress, body temperature, or medication, all of which likely play a role in pwMS (40). In addition, it is important to be aware of possible alterations in cardiovascular autonomic control in some pwMS (41). A delayed heart rate response during high-intensity activities, especially those of short duration, might have resulted in lower estimates of physiological strain (42). An alternative measurement might be oxygen uptake during societal participation. Furthermore, heart rate measured by a Fitbit might have resulted in an underestimation of physiological strain compared with heart rate measured by gold standard ECG measurements (29). A more accurate approach might involve ECG or a chest-worn heart rate monitor. Moreover, we estimated HRmax of participants using the Karvonen formula. This might have underestimated the HRmax, and to a lesser extent also underestimated physiological strain measured as %HRR, especially in the older study population (43). Finally, perceived strain was measured on a scale of 1 to 10, while exercise studies usually use 0–10 or 6–20 scales. The 1–10 scale used reduces generalizability and makes comparisons of studies difficult (35). For future studies it would be insightful to add a healthy control group. Including a healthy control group can provide insight into the relationship between perceived and physiological strain among healthy people and whether this differs between healthy controls and pwMS.

### Conclusion

Assessment of the 7-day relationship between patient-perceived and physiological strains during societal participation in pwMS showed that the perceived and physiological strain of societal participation should be considered as 2 different constructs. The Whereabouts smartphone app provided valuable information to users concerning perceived strains, a factor that may limit active societal participation. We suggest that future measurement of the physiological strains of societal participation will require the deployment of additional wearables.
